# Analysis of vasovagal syncope in the blood collection room in patients undergoing phlebotomy

**DOI:** 10.1038/s41598-020-74265-9

**Published:** 2020-10-21

**Authors:** Akira Yoshimoto, Atsushi Yasumoto, Yuko Kamiichi, Haruna Shibayama, Masaya Sato, Yoshiki Misawa, Kazuharu Morita, Yoshikazu Ono, Shinji Sone, Tomoaki Satoh, Yutaka Yatomi

**Affiliations:** 1 Department of Clinical Laboratory, The University of Tokyo Hospital, 7-3-1 Hongo, Bunkyo-ku, Tokyo, 113-8655 Japan; 2 International University of Health and Welfare Mita Hospital, 1-4-3 Mita, Minato-ku, Tokyo, 108-8329 Japan

**Keywords:** Health care, Medical research, Signs and symptoms

## Abstract

Vasovagal syncope (VVS) is well-known to occur in patients undergoing phlebotomy, however, there have been no large-scale studies of the incidence of VVS in the blood collection room. The aim of our present retrospective study was to investigate the conditions of phlebotomy and determine the incidence/factors predisposing to the development of VVS. We investigated 677,956 phlebotomies performed in outpatients in the blood collection room, to explore factors predisposing to the development of VVS. Our analysis revealed an overall incidence of VVS of 0.004% and suggested that use of more than 5 blood collection tubes and a waiting time of more than 15 min were associated with a higher risk of VVS. The odds ratios of these factors were 8.10 (95% CI 3.76–17.50) and 3.69 (95% CI 0.87–15.60), respectively. This is the large-scale study to analyze factors of the development of VVS in the blood collection room, and according to our results, use of a large number of blood collection tubes and a prolonged waiting time for phlebotomy may be risk factors for the development of VVS.

## Introduction

Vasovagal reactions (VVRs) are encountered in blood donors at blood centers, manifesting with symptoms such as pallor, perspiration, dizziness, nausea, and fainting^1^. Young age, female gender, first experience, and low-body weight have been reported as risk factors for the development of VVRs, and VVRs during blood donation have been reported to occur at a frequency of 2–5%^2–4^. It seems likely that the incidence of VVRs in blood donors is higher than that in phlebotomy patients probably due to the difference of blood volume collected. In addition, fear of venipuncture and the time taken to collect blood have been reported to be associated with a higher incidence of VVRs^5^. These reports concluded that both biological factors and psychological stress are important factors influencing the incidence of VVRs during blood donation^2–5^.

VVRs are also known to occur in patients undergoing phlebotomy for blood sampling at hospitals, although the frequency is quite low. Little research has been conducted to determine the incidence of VVRs, much less vasovagal syncope (VVS), in patients undergoing phlebotomies at hospitals, although the reported incidence rates of VVRs and VVS from a survey of three hospital-based outpatient phlebotomy clinics were 0.4% and 0.2%, respectively^6^. When one patient develops a VVS in the blood collection room, some phlebotomists would take care of the patient, which could delay the blood collection of the next and all subsequent patients, that is, an overall delay in the turnaround time. In order to prevent the development of VVS and maintain the safety of patients in the blood collection room, we were prompted to analyze the factors predisposing to the development of VVS. Also, a large sample size would be needed, because the incidence of VVS in patients undergoing phlebotomy is extremely low.

The aim of our retrospective study was to investigate the phlebotomy conditions of outpatients undergoing phlebotomy in the blood collection room of our hospital and determine the incidence/factors predisposing to the development of VVS. To the best of our knowledge, there have been no reports of any large-scale study on the incidence and causes of VVS in the blood collection room of a large hospital, and this is the first report.

## Methods

### Patient recruitment

All outpatients who underwent phlebotomy at the blood collection room of The University of Tokyo Hospital between January 1, 2015, and December 31, 2017, were enrolled in this study. VVS was defined as an abrupt, transient, complete loss of consciousness associated with an inability to maintain the postural tone, with rapid and spontaneous recovery^7^. Cases of immediate VVS, occurring at blood collection room immediately after phlebotomy, were enrolled. Patients suffering from hypoglycemia, epileptic stroke, narcolepsy, hyperventilation syndrome or hysterical attack were not labeled as having developed VVS. Informed consent for participation in the study was obtained using an opt-out process on the web page of The University of Tokyo Hospital. This study was conducted with the approval of the ethics committee of The University of Tokyo Hospital (No. 11804), in compliance with the relevant guidelines and regulations.

### Blood collection conditions

The subjects were outpatients who were more than 2 years old from all clinical departments; their conditions could not be fully identified and it was not clear whether they were fasting or not before their phlebotomies because of the retrospective study. The phlebotomies were performed with patients being in a sitting position on a chair. We obtained information from the blood collection system (Techno Medica Co., Tokyo) about the time of arrival of the subject at the reception of the blood collection room and the time at which he/she was called to the blood collection booth for the phlebotomy. From these times, the waiting time was calculated for each patient. Moreover, the number of waiting patients and the ratio of the number of waiting patients to the number of functioning blood collection booths at the reception were analyzed as indicators of the degree of crowding in the blood collection room.

We evaluated the incidence of VVS by the time zone of phlebotomy, number of blood collection tubes, waiting time, number of waiting patients, and the ratio of the number of waiting patients to the number of functioning blood collection booths. In addition, the condition in the present phlebotomy experience associated with the development of VVS (VVS (+) phlebotomy experience) and that in past phlebotomy experience not associated with the development of VVS (VVS (−) phlebotomy experience) were compared in the same patients.

### Statistical analysis

All statistical analyses were performed with EZR (Saitama Medical Center, Jichi Medical University, Saitama, Japan), which is a graphical user interface for R (The R Foundation for Statistical Computing, Vienna, Austria). More precisely, it is a modified version of R commander designed to add statistical functions frequently used in biostatistics^8^. The Chi-square test was used for analysis of the incidence of VVS. Logistic regression analysis was performed to determine the association of VVS with the risk factors that were identified by the Chi-square test as being significant. Wilcoxon’s signed-rank test was used for comparisons of the conditions in the present VVS (+) phlebotomy experience and that in past VVS (−) phlebotomy experience in the same patients. Values are expressed as the medians, and p < 0.05 were defined as being indicative of statistically significant difference.

## Results

### Incidence of VVS among outpatients

**Figure 1:**
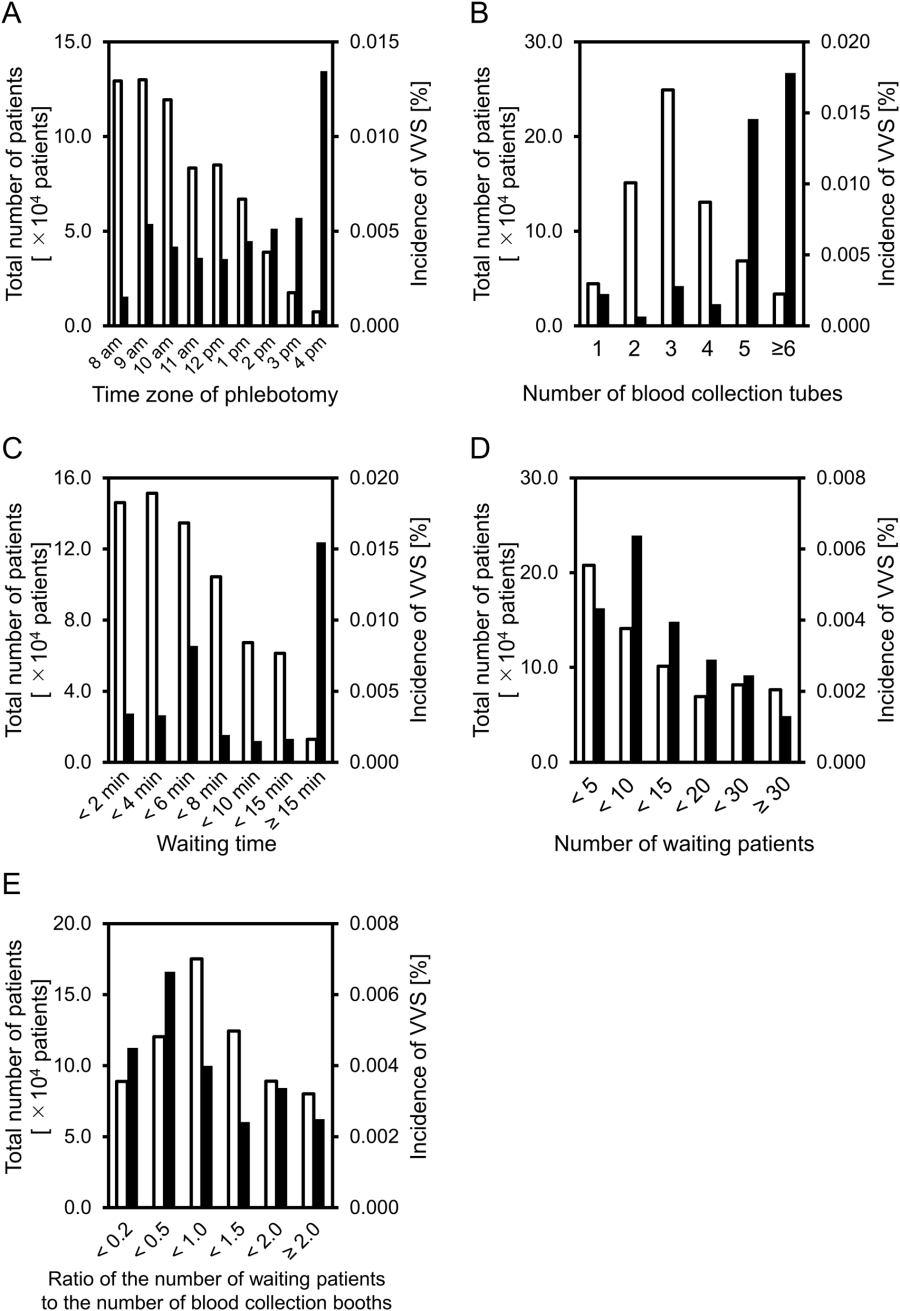
Incidence of VVS. The data of patients who had undergone phlebotomy at the blood collection room of our hospital between January 1, 2015, and December 31, 2017 (n = 677,956) were analyzed. The incidence of VVS was evaluated by the time zone of the day in which the phlebotomies were performed (**A**), the number of blood collection tubes used (**B**), the waiting time (**C**), the number of waiting patients (**D**), and ratio of the number of waiting patients to the number of functioning blood collection booths (**E**). The clear and filled bar graphs show the total number of patients and the incidence of VVS, respectively.

There are 18 blood collection booths for outpatients in the blood collection room of our hospital, and about 1000 outpatients undergo phlebotomy in this room every day. The total number of patients enrolled in this study was 677,956, and the number (percentage) of patients who developed VVS was 27 (0.004%). The waiting time for phlebotomy was less than 10 min for ≥ 90% of patients. Of 27 VVS patients, 19 (70%) were female and 8 (30%) were male. Fifteen (56%) patients experienced VVS at the first phlebotomy in our hospital. The median age of VVS patients was 23 years old, and the number of VVS patients was the largest in 20–29 years old group (Table 1). In regard to the time zone of phlebotomy, the incidence of VVS was the highest in the phlebotomies conducted in the time zone of 4–5 pm (1/7433; 0.013%), and around 0.004% in phlebotomies conducted in other time zones (Fig. 1A). Analysis conducted by the number of blood collection tubes used revealed that the incidence of VVS was higher in patients for whom 5 (10/68,653; 0.015%) and more than 6 blood collection tubes (6/33,683; 0.018%) were needed, while that in the patients for whom 3 tubes were used (the predominant patient group) was 0.003% (7/249,265) (Fig. 1B). The volumes of blood withdrawn for 1, 2, 3, 4, 5, and more than 6 tubes were 4.1 ± 1.4, 6.6 ± 0.9, 8.6 ± 1.2, 11.3 ± 1.9, 14.3 ± 2.4, 19.9 ± 4.5 mL (mean ± SD), respectively. Next, comparison of the incidences of VVS by the waiting time revealed that the incidence was the highest in the patient group with waiting times for the phlebotomy of more than 15 min (2/12,936; 0.015%) (Fig. 1C). Finally, comparison of the incidence of VVS by the number of waiting patients and ratio of the number of waiting patients to the number of functioning blood collection booths revealed that the incidence of VVS was around 0.004% in each group (Fig. 1D,E).

**Table 1:** Characteristics of VVS patients.

	Male	Female	Total
N	8	19	27
**Age**			
2–19	2	5	7
20–29	4	7	11
30–39	0	1	1
40–49	1	3	4
50–59	0	2	2
60–99	1	1	2
**Past experience of phlebotomy**			
0	4	11	15
≥ 1	4	8	12

*VVS* vasovagal syncope.

The incidence of VVS was significantly different depending on the number of blood collection tubes used (*df* = 5, *X*^*2*^ = 43, *p* < 0.0001) and the waiting time for the phlebotomy (*df* = 8, *X*^*2*^ = 14, *p* < 0.05); on the other hand, no significant differences were noted depending on the time zone of phlebotomy, number of waiting patients in the blood collection room, or the ratio of the number of waiting patients to the number of functioning blood collection booths (Supplementary Table S1). The odds ratio of use of more than 5 blood collection tubes for the development of VVS was 8.10 (95% CI 3.76–17.50, p < 0.001), indicating that a large number of blood collection tubes used may predispose to the development of VVS in the blood collection room for outpatients (Table 2). The odds ratio of a waiting time of more than 15 min for the phlebotomy for the development of VVS was 3.69 (95% CI 0.87–15.60, p = 0.076); although this ratio was not significant, the high odds ratio implies the possibility that a prolonged waiting time may also contribute to the risk of development of VVS.

**Table 2:** Logistic regression analysis to identify the risk factors for VVS.

Phlebotomy		VVS		Odds ratio	95% CI for OR		P-value
	Number	Number	%	(OR)	Lower	Upper	
**Number of blood collection tubes**							
≥ 5	102,336	16	0.016	8.10	3.76	17.50	0.0000
< 5	575,620	11	0.002	1.00			
**Waiting time (min)**							
≥ 15	13,252	2	0.015	3.69	0.87	15.60	0.0757
< 15	664,704	25	0.004	1.00			

*VVS* vasovagal syncope.

### The relationship between the incidence of VVS and the phlebotomy conditions in the same patients

**Figure 2:**
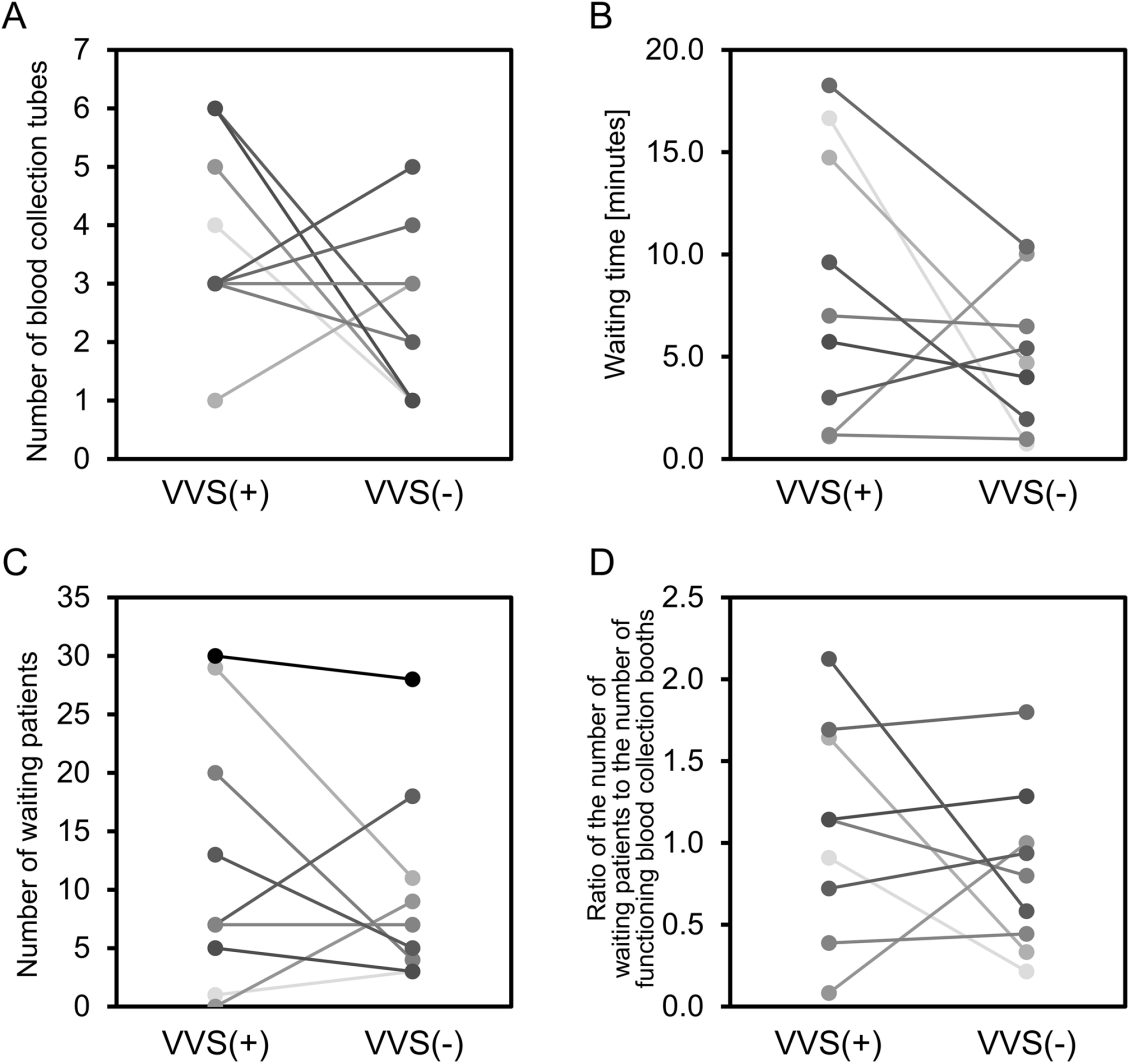
Comparisons of the conditions in the present phlebotomy experience associated with the development of VVS (VVS (+) phlebotomy experience) and past phlebotomy experience not associated with VVS (VVS (−) phlebotomy experience) in the same patients. Number of blood collection tubes (**A**), waiting time (**B**), number of waiting patients (**C**), and ratio of the number of waiting patients to the number of functioning blood collection booths (**D**) were compared between the present phlebotomy experience associated with the development of VVS (VVS (+) phlebotomy experience) and past phlebotomy experience not associated with VVS (VVS (−) phlebotomy experience) in the same patients (n = 9). Each line shows the data of individual patients.

To further examine the relationship between the incidence of VVS and the phlebotomy conditions under the same biological conditions, the conditions in the present phlebotomy experience associated with the development of a VVS (VVS (+) phlebotomy experience) and past phlebotomy experience (within 6 months) not associated with the development of VVS (VVS (−) phlebotomy experience) were compared in the same patients (n = 9) (Fig. 2). There was no significant difference in the number of blood collection tubes (3 vs. 2, p = 0.18), waiting time (7.0 min vs. 4.7 min, p = 0.17), number of waiting patients (7 vs. 7, p = 0.48), or the ratio of the number of waiting patients to the number of functioning blood collection booths (1.1 vs. 0.8, p = 0.51) between the VVS (+) phlebotomy experience and VVS (−) phlebotomy experience.

## Discussion

### The number of blood collection tubes and waiting time as risk factors of VVS

We have found the risk factors of VVS in phlebotomy, i.e., the number of blood collection tubes and waiting time in the present study. The incidence of VVS was higher in phlebotomies in which more than 5 blood collection tubes were used and the waiting time for the phlebotomy was more than 15 min; in particular, use of more than 5 blood collection tubes appeared to be a significant risk factor for the development of VVS. We will consider the risk factor in detail below.

### The comparison of the phlebotomies with and without the development of VVS

Since previous reports have suggested that the development of VVRs could be related to biological factors such as the age, sex, history of phlebotomies, and body weight^3–5^, we investigated the relationship between the incidence of VVS and the phlebotomy conditions after adjustments for these biological factors. In this retrospective study, we compared the conditions of the present VVS (+) phlebotomy experience and past VVS (−) phlebotomy experience in the same patients. The results showed the absence of any significant differences in the phlebotomy conditions between the present VVS (+) phlebotomy experience and past VVS (−) phlebotomy experience, suggesting that the incidence of VVS was related to factors other than the phlebotomy conditions. Thus, both biological factors and phlebotomy conditions, especially use of more than 5 blood collection tubes and a waiting time of more than 15 min may be associated with an increased risk of development of VVS. Since information on biological factors, such as the age, sex, and body weight, of the subjects was not available in the present study, further studies are needed to investigate the relationships between biological factors and the risk of development of VVS during phlebotomies.

### The incidence of VVS associated with phlebotomy

The incidence of VVS in our study, i.e., 0.004%, was much lower than previously reported data, i.e., 2–5%^2–4^. The subjects in our study underwent phlebotomy for clinical laboratory tests, in which the volume of blood for phlebotomy (4–20 mL) was smaller than that for donation (500 mL)^4^. The similar study to ours reported the low incidence, i.e., 0.2%^6^ while most studies on the subjects undergoing blood donation reported higher incidence of VVS. VVS was reported to develop at the frequency of 2.5% during blood sampling tests^9^, in which the subjects analyzed were much younger, which might result in the higher incidence of VVS, than those of our study. We speculate that employment of older people undergoing phlebotomy for clinical laboratory tests might have resulted in extremely lower incidence of VVS in our study although further analysis may be required. Furthermore, we evaluated only ‘immediate’ form VVS (i.e., before departure), but not ‘delayed’ form (i.e., after leaving but typically less than 24 h), which may account for the low event rate in our study at least partly.

### The risk factors of VVS associated with phlebotomy

According to a study on blood donation, the perceived volume of blood loss was related to the development of VVS^10^. The number of blood collection tubes used could be considered to correspond to the perceived volume of blood loss. Thus, the higher incidence of VVS associated with the use of a large number of blood collection tubes in our study could be considered as being due to the perceived large volume of blood loss associated with the use of a large number of collection tubes. The fear of venipuncture has been reported to be associated with an increased risk of VVS; that is, psychological stress is an important factor predisposing to the development of VVS^5^. Moreover, it is possible that a large volume of blood drawn and a prolonged waiting time could interact to increase the risk of development of VVS, because both fear and a long blood sampling time can increase the risk of VVS and the risk could be additive^5^. Therefore, a prolonged waiting time and/or use of a large number of blood collection tubes could increase the psychological stress and predispose to the development of VVS.

The waiting time for phlebotomies has recently attracted attention in studies of blood collection rooms. The waiting time has been reported to be significantly associated with the overall satisfaction of patients toward clinical laboratory services^11^. It has also been reported that introduction of a new system in the blood collection room has successfully shortened the waiting time and decreased the turnaround time in blood collection rooms^12^. Taken together, shortening of the waiting time is expected to not only contribute to decrease of the turnaround time, but also to a higher satisfaction level of the patients, which underscores the importance of shortening the waiting time. Our study provides new insight into the importance of waiting time as a useful target for the prevention of VVS.

## Limitation

We could not obtain information on the patient characteristics, such as the age, sex, and body weight, in the present study, because our blood collection system did not indicate personal information of patients in detail. Although we could investigate information on the VVS patient using medical records, further study was needed for demographic data about all patients who receive phlebotomy service.

## Conclusion

In conclusion, we found that use of a large number of blood collection tubes was associated with a higher incidence of VVS in the blood collection rooms for outpatients. A prolonged waiting time also appeared to be associated with a higher risk of development of VVS. Therefore, the reduction of the number of blood collection tubes and the shortage of waiting time would prevent the development of VVS in a blood collection room.

## Supplementary information

Supplementary Table S1.
