# Supplementary material for: Analysis of vasovagal syncope in the blood collection room in patients undergoing phlebotomy
Source: Sci Rep. 2020 Oct 21;10:17933. doi: 10.1038/s41598-020-74265-9 (PMC7578787; doi:10.1038/s41598-020-74265-9)
Supplement: Supplementary file 1 — Supplementary Table S1. [file 41598_2020_74265_MOESM1_ESM.docx]

**Analysis of vasovagal syncope in the blood collection room in patients undergoing phlebotomy**

Akira Yoshimoto^1^, Atsushi Yasumoto^1^, Yuko Kamiichi^1^, Haruna Shibayama^1^, Masaya Sato^1^, Yoshiki Misawa^1^, Kazuharu Morita^1^, Yoshikazu Ono^1^, Shinji Sone^2^, Tomoaki Satoh^1^ and Yutaka Yatomi^1^

^1^Department of Clinical Laboratory, The University of Tokyo Hospital, 7-3-1 Hongo, Bunkyo-ku, Tokyo 113-8655, Japan

^2^International University of Health and Welfare Mita Hospital, 1-4-3 Mita, Minato-ku, Tokyo 108-8329, Japan

Supplementary Table S1. Analysis of incidence of vasovagal syncope

|  |  |  | Patients |  | VVS | | |  | Chi-square test |
| --- | --- | --- | --- | --- | --- | --- | --- | --- | --- |
|  |  |  | Number |  | Number |  | % |  | *P-value* |
| Time zone of phlebotomy | | | |  |  |  |  |  | 0.7957 |
|  | 8:00 AM |  | 129385 |  | 2 |  | 0.002 |  |  |
|  | 9:00 AM |  | 129992 |  | 7 |  | 0.005 |  |  |
|  | 10:00 AM |  | 119410 |  | 5 |  | 0.004 |  |  |
|  | 11:00 AM |  | 83356 |  | 3 |  | 0.004 |  |  |
|  | 12:00 PM |  | 84982 |  | 3 |  | 0.004 |  |  |
|  | 1:00 PM |  | 66947 |  | 3 |  | 0.004 |  |  |
|  | 2:00 PM |  | 38921 |  | 2 |  | 0.005 |  |  |
|  | 3:00 PM |  | 17530 |  | 1 |  | 0.006 |  |  |
|  | 4:00 PM |  | 7433 |  | 1 |  | 0.013 |  |  |
| Number of blood collection tubes | | | | | | | |  | < 0.0001 |
|  | 1 |  | 44549 |  | 1 |  | 0.002 |  |  |
|  | 2 |  | 151098 |  | 1 |  | 0.001 |  |  |
|  | 3 |  | 249265 |  | 7 |  | 0.003 |  |  |
|  | 4 |  | 130708 |  | 2 |  | 0.002 |  |  |
|  | 5 |  | 68653 |  | 10 |  | 0.015 |  |  |
|  | ≥6 |  | 33683 |  | 6 |  | 0.018 |  |  |
| Waiting time | |  |  |  |  |  |  |  | 0.0356 |
|  | < 2 min |  | 146083 |  | 5 |  | 0.003 |  |  |
|  | < 4 min |  | 151388 |  | 5 |  | 0.003 |  |  |
|  | < 6 min |  | 134689 |  | 11 |  | 0.008 |  |  |
|  | < 8 min |  | 104357 |  | 2 |  | 0.002 |  |  |
|  | < 10 min |  | 67292 |  | 1 |  | 0.001 |  |  |
|  | < 15 min |  | 61211 |  | 1 |  | 0.002 |  |  |
|  | ≥ 15 min |  | 12936 |  | 2 |  | 0.015 |  |  |
| Number of waiting patients | | | | | | | |  | 0.5895 |
|  | < 5 |  | 207781 |  | 9 |  | 0.004 |  |  |
|  | < 10 |  | 141072 |  | 9 |  | 0.006 |  |  |
|  | < 15 |  | 101215 |  | 4 |  | 0.004 |  |  |
|  | < 20 |  | 69348 |  | 2 |  | 0.003 |  |  |
|  | < 30 |  | 81877 |  | 2 |  | 0.002 |  |  |
|  | ≥ 30 |  | 76663 |  | 1 |  | 0.001 |  |  |
| Ratio of the number of waiting patients  to the number of functioning blood collection booths | | | | | | | | | 0.6226 |
|  | < 0.2 |  | 88849 |  | 4 |  | 0.005 |  |  |
|  | < 0.5 |  | 120362 |  | 8 |  | 0.007 |  |  |
|  | < 1.0 |  | 175168 |  | 7 |  | 0.004 |  |  |
|  | < 1.5 |  | 124355 |  | 3 |  | 0.002 |  |  |
|  | < 2.0 |  | 89032 |  | 3 |  | 0.003 |  |  |
|  | ≥ 2.0 |  | 80190 |  | 2 |  | 0.002 |  |  |

VVS: Vasovagal syncope
